# Analysis of a case report of meningitis caused by *Listeria monocytogenes*

**DOI:** 10.3389/fmed.2024.1440225

**Published:** 2024-09-11

**Authors:** Lihui Chen, Manman Pei, Xingxing Wang, Yongfeng Zhang, Yuquan Ma, Yifei Chen, Ishtiaq Ahmad

**Affiliations:** ^1^Department of Laboratory, Jincheng People's Hospital, Jincheng, Shanxi, China; ^2^Department of Global Health Research, Graduate School of Medicine, Juntendo University, Bunkyo-ku, Japan

**Keywords:** *Listeria monocytogenes*, foodborne, meningitis, antibiotic therapy, case report

## Abstract

**Background:**

*Listeria monocytogenes* is a Gram-positive bacterium transmitted to humans through contaminated food, water, and animal faeces, posing a public health risk. *Listeria monocytogenes* is difficult to isolate and is not sensitive to first-line treatment with broad-spectrum cephalosporins for bacterial meningitis. Listeria meningitis is rare but can progress rapidly and may be accompanied by serious complications (hydrocephalus, ventricular inflammation, cerebral palsy, and brain abscess) and a high mortality rate.

**Case presentation:**

It is a retrospective analysis of the clinical characteristics and treatment of a rare case of *Listeria monocytogenes* infection. Using laboratory indicators such as white blood cells (WBC), C-reactive protein (CRP), and procalcitonin (PCT), three detection methods (cerebrospinal fluid/blood culture), Targeted gene sequencing technology (tNGS), and Metagenomic next-generation sequencing technology (mNGS) combined with clinical manifestations of patients, analyze the use plan and prognosis of antibiotics in patients. The patient in this case initially had neurological symptoms such as fever, headache, unclear consciousness, and vomiting; laboratory indicators include elevated WBC, CRP, and PCT. *Listeria monocytogenes* was cultured in both the patient’s cerebrospinal fluid and blood samples. After treatment with penicillin and meropenem, the patient recovered and was discharged without any sequelae.

**Conclusion:**

Due to the rarity of *Listeria monocytogenes*, there may be deficiencies and difficulties in clinical differential diagnosis, making it difficult to achieve targeted antibiotic treatment. Therefore, accurate identification of *Listeria monocytogenes* and relevant laboratory inflammation indicator testing, combined with traditional culture methods and NGS testing, through empirical coverage of *Listeria monocytogenes*, targeted antibiotic treatment ultimately impacts clinical outcomes significantly.

## 1 Introduction

*Listeria monocytogenes* (LM) is a Gram positive, spore free, short rod-shaped bacterium whose colonies are catalase positive when grown on blood agar, the disease caused by this bacterium is called human listeriosis (1). The incidence of human listeriosis in the European Union from 2014 to 2018 ranged from 0.43 to 0.48 cases per 100,000 people (1). Although its incidence rate is low, it is rare (2), and the mortality of listeriosis caused by it is as high as 20% ~ 30% (3). In 2015, a report of 2,224 cases of LM infection in Europe showed a total mortality rate of 18.8%. From 2011 to 2016, 19 provinces in China reported 253 cases of LM infection, with a mortality rate of 25.7% (4).

LM can cross the blood–brain barrier and cause intracranial infection, which leads to meningitis. Reducing the incidence rate and mortality of meningitis is the focus of the WHO Global Roadmap to Combat Meningitis by 2030 (4). Meningitis caused by LM has a dangerous onset, high mortality rate, and atypical clinical symptoms. Due to its high salt tolerance, wide pH and temperature range, and ability to grow and reproduce under 4°C conditions, this bacterium can be obtained in refrigerated food, causing LM meningitis and posing a threat to human health (5). The diagnosis of *Listeria monocytogenes* requires the cultivation of LM bacteria in blood or cerebrospinal fluid. Due to the rarity of this bacterium and the atypical clinical symptoms, the early diagnosis and precise treatment of LM infection still face significant challenges. In recent years, with the continuous progress of cerebrospinal fluid pathogen diagnosis technology, the positive rate of meningitis pathogen diagnosis has been continuously increasing. Next-generation gene sequencing (tNGS) is another sequencing method used for clinical microbial identification in addition to Metagenomic next-generation sequencing technology (mNGS), which is mostly not affected by the human genome and colonizing bacteria. It is effective in identifying a low copy number of infectious diseases that are widely known and widely spread. In this case, Targeted gene sequencing technology (tNGS) is used to monitor patient outcomes after treatment, while mNGS is used to identify LM patients (6).

In summary, these three methods provide sufficient etiological evidence to prove the rationality of the treatment plan and provide new ideas for the diagnosis of LM. Due to the relatively small number of cases of LM infection meningitis, there are not many reports both domestically and internationally. This article explores the clinical characteristics, treatment, and prognosis of a patient with LM infection meningitis.

## 2 Case description

### 2.1 Medical history

**Figure 1:**
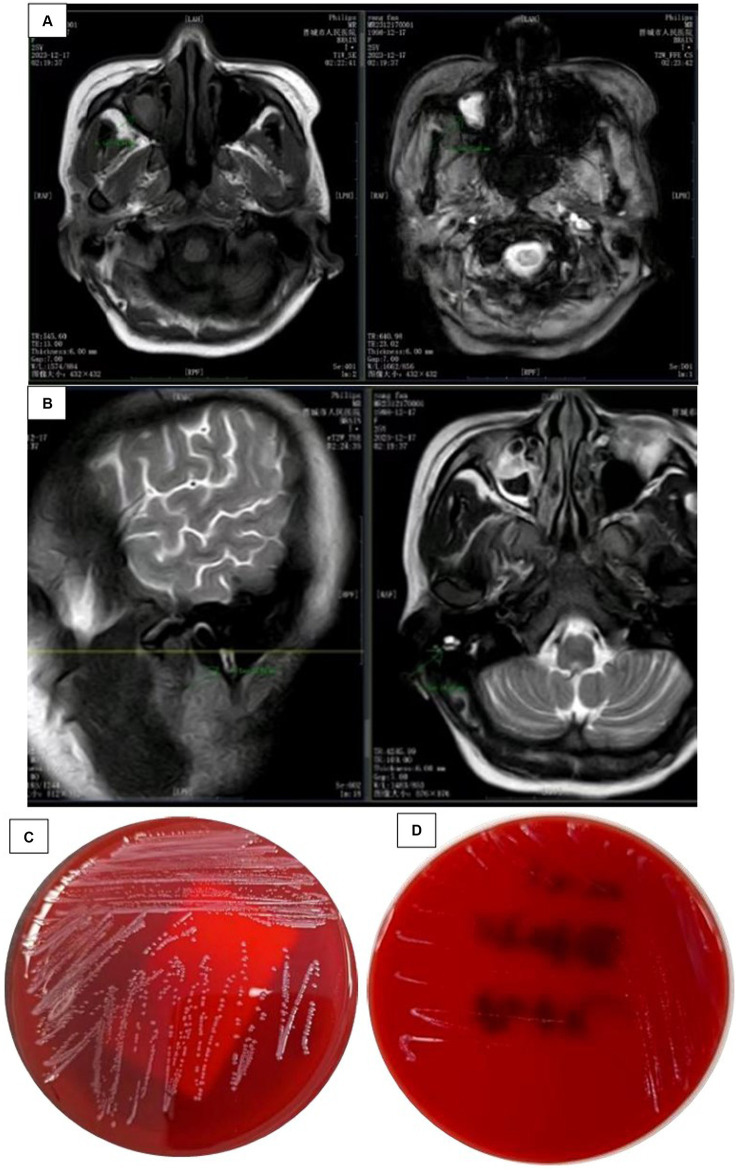
**(A)** Head MRI findings: a plain scan of the head MRI shows obvious abnormalities, with rig maxillary sinusitis (excluding fungal lesions) and **(B)** Head MRI findings: a plain scan of the hea MRI shows significant abnormalities, with MR changes in right mastoiditis. **(C)** Cultured CSI colonies and **(D)** the growth of bacterial colonies in the refrigerator.

The patient, a young female, was admitted to the hospital mainly due to “headache and fever for 2 days, unclear consciousness for 1 day.” Two days ago, she developed a headache on both sides of the temporal and occipital regions, which appeared to be distressing and persistent, accompanied by fever, no nausea and vomiting, and no limb convulsions. Later, the headache worsened, followed by unclear consciousness, inability to respond, obvious irritability, shouting, and urinary incontinence. She urgently sought medical attention at our hospital and underwent a complete head MRI scan, which showed no obvious abnormalities. Right maxillary sinusitis (excluding fungal infections) was found on the right side of the MRI, and there were no obvious abnormalities on the right side of the MRI. Mastitis MR changes (Figures 1A,B), improved lumbar puncture surgery, “pressure greater than 330mmH_2_O, cerebrospinal fluid routine shows: white blood cell count 2.797 × 10^9^/L, percentage of mononuclear cells 66%, cerebrospinal fluid biochemistry shows: protein 1.91 g/L, chloride 113.0 mmol/L, sugar 0.7 mmol/L,” blood routine shows white blood cells 24.77 × 10^9/^L, red blood cells 2.04 × 10^12^/L, hemoglobin 53 g/L, C-reactive protein 43.04 mg/L, Determination of D-dimer content at 1.28 mg/L. Emergency biochemical sodium 126.5 mmol/L, chlorine 94.7 mmol/L. Erythrocyte sedimentation rate (ESR) 86 mm/H.

**Figure 2:**
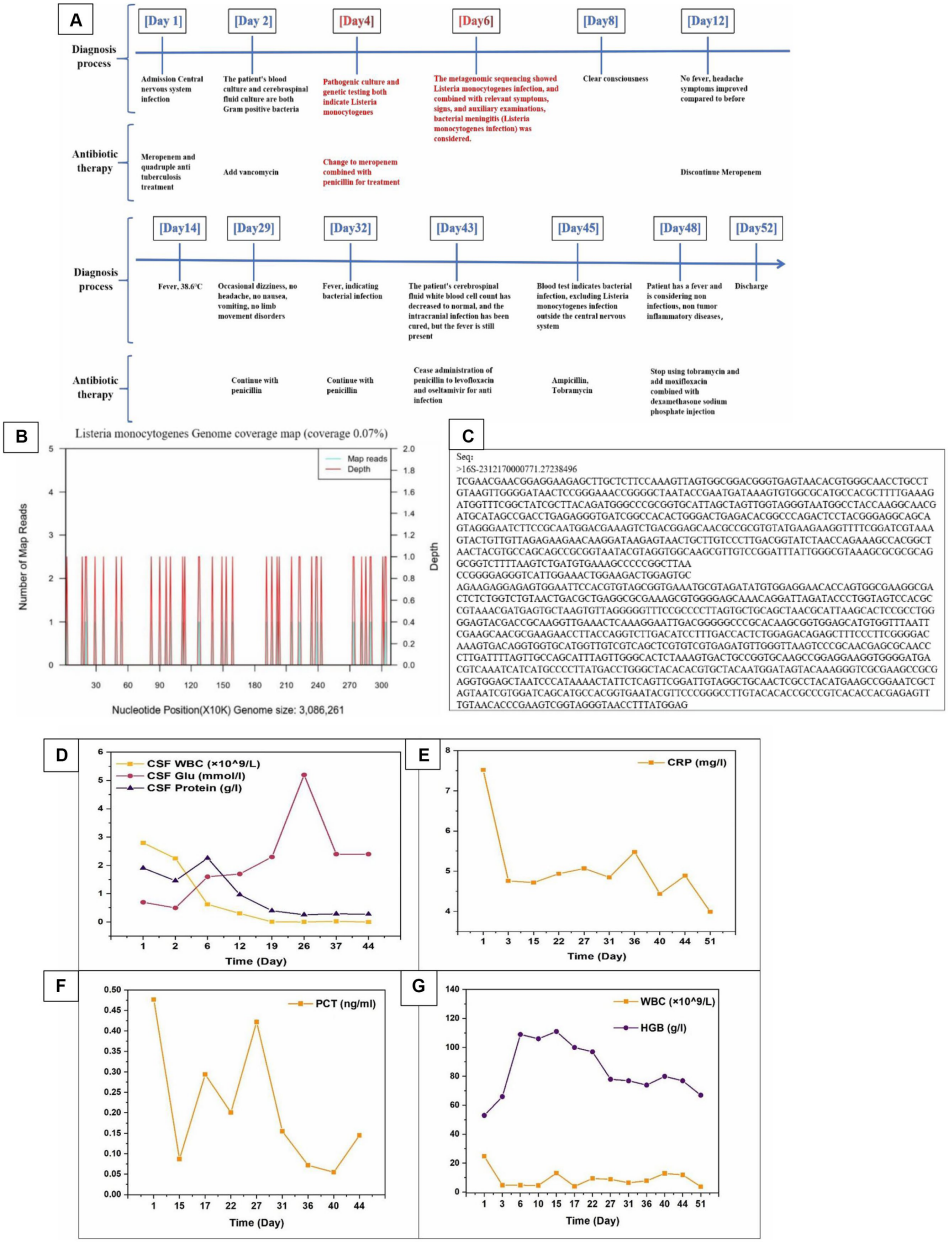
**(A)** The diagnosis and treatment process of patients after admission. **(B)** The genomic coverage map of *Listeria monocytogenes* in the identification results of mNGS for cerebrospinal fluid metagenomes. **(C)** Targeted DNA sequencing. **(D–G)** The relevant laboratory examination indicators of patients during hospitalization (CSF WBC, CSF Glu, CSF Protein, CRP, PCT, WBC, and HGB).

Since the onset of the disease, She has been confused, irritable, and experiencing urinary incontinence. Diagnosed with a central nervous system infection and admitted to our hospital. The diagnosis and treatment process of the patient after admission is shown in Figure 2A.

### 2.2 History and personal history

The patient was previously diagnosed with adult Still’s disease due to fever, rash, etc., and has been treated with oral steroids, cyclosporine, methotrexate, etc. During the visit, miliary pulmonary tuberculosis was found, and standardized anti-tuberculosis treatment improved nearly a year later. Intermittent fever occurred again in March 2023, which could spontaneously subside. In September, Targeted gene sequencing of alveolar lavage fluid revealed *Mycobacterium tuberculosis*. It was recommended to seek treatment at a specialized hospital, but the effect was poor and the patient was discharged from the hospital. Anaemia for 4 years, with a history of blood transfusion. No history of living in another city, no smoking history, no history of exposure to toxins.

### 2.3 Vital signs upon admission

Body temperature 38.9°C, pulse 133 times/min, respiration 27 times/min, blood pressure 136/82 mmHg (1 mmHg = 0.133 kPa). Auscultation of both lungs revealed rough breathing sounds, no wet rales, and no pleural friction sounds. Physical examination of the heart and abdomen showed no abnormalities. Neurological specialist physical examination: unclear consciousness, poor mental state, irritability, shouting loudly, aconuresis. Bilateral pupils are equally large and round, with a diameter of approximately 3.0 mm, and are sensitive to light reflection. All limbs can be lifted off the bed surface, with slightly higher muscle tone, symmetrical tendon reflexes on both sides and positive pathological signs on both sides. Neck resistance, four transverse fingers under the chin, Kerning sign (+), Brudzinski sign (+). Other physical examinations cannot be coordinated.

### 2.4 Auxiliary examination

Hepatitis virus, syphilis, and human immunodeficiency virus (HIV) are all normal. General bacterial and fungal smear-negative, meningococcal smear negative; Cryptococcus smear negative; The tuberculosis smear is negative.

### 2.5 Treatment and prognosis

Rescue records show that the patient is considering severe encephalitis, which is a critical condition with high risk and may result in sequelae such as encephalitis. Meropenem was administered for anti-infective treatment, along with isoniazid, rifampicin, ethambutol, and pyrazinamide as a quadruple anti-tuberculosis treatment. On the second day of admission, the critical value of the laboratory was received: the patient’s blood culture and cerebrospinal fluid culture showed bacterial growth as Gram-positive bacteria (Figure 1C), and the colony was selected for cold inoculation in the refrigerator (Figure 1D). Head CT and chest CT display: No obvious abnormalities were found on the plain scan of the head CT, with double pneumonia lesions. It is recommended to have a follow-up examination after treatment, with a small amount of pericardial effusion; Cardiac ultrasound and electrocardiogram showed no abnormalities. Based on clinical manifestations and bacterial culture results, a preliminary diagnosis of bacterial meningitis is made. The patient had a severe intracranial infection and was treated with meropenem 2 g, once/8 h, combined with vancomycin 1 g, once/12 h, intravenous drip of antibacterial therapy, and mannitol 250 mL: 50 g, once/6 h. The patient showed significant restlessness and was given midazolam and diazepam sedation, as well as quetiapine antipsychotic treatment. To correct anaemia, 400 mL of positive white blood cell suspension red blood cells were infused.

On the 3rd day of admission, the blood white blood cell count was rechecked to be 4.79 × 10^9^/L, the absolute value of monocytes was 0.74 × 10^9^/L, and the percentage of monocytes was 14.5%; C-reactive protein 60.7 mg/L, blood electrolyte sodium 132.3 mmol/L, electrolyte chlorine 102.8 mmol/L, red blood cell count 2.35 × 10^12^/L, hemoglobin 66 g/L. The patient’s restlessness symptoms improved and there was no further fever. Resuspended red blood cell infusion to correct anaemia.

On the 4th day of admission, the identification results of cerebrospinal fluid culture (Table 1) and blood culture (Table 2) were both *Listeria monocytogenes* and the drug sensitivity results were sensitive to penicillin and meropenem.

**Table 1:** The identification results of blood culture.

**Identification information**	**Card:** GP	**Lot Number:** 2422394503	**Expires:** Jun 16, 2024 12:00 CST
	**Status:** Final	**Analysis Time:** 6.98 h	**Completed:** Dec 19, 2023 17:44 CST
**Organism origin**	VITEK 2		
**Selected organism**	99% Probability *Listeria monocytogenes* Bionumber: 342200224733621 Confidence: Excellent identification		
**Analysis organisms and tests to separate:**			
**Analysis messages:** Critical pathogen, check camp test and beta-hemolysis			
**Contraindicating typical biopattern(s)**			

**Table 2:** The identification results of cerebrospinal fluid culture.

**Identification information**	**Card:** GP	**Lot Number:** 2422394503	**Expires:** Jun 16, 2024 12:00 CST
	**Status:** Final	**Analysis Time:** 6.97 h	**Completed:** Dec 19, 2023 17:43 CST
**Organism origin**	VITEK 2		
**Selected organism**	99% Probability *Listeria monocytogenes* Bionumber: 342200224733621 Confidence: Excellent identification		
**Analysis organisms and tests to separate:**			
**Analysis messages:** Critical pathogen, check camp test and beta-hemolysis			
**Contraindicating typical biopattern(s)**			

Targeted gene sequencing of cerebrospinal fluid identified genus Listeria (Table 3), and the Metagenomic next-generation sequencing technology results of cerebrospinal fluid external examination (Zhengzhou Jinyu Main Laboratory) also showed *Listeria monocytogenes* (Table 4; Figure 2B). Due to the genetic similarity between *Listeria monocytogenes* and *Listeria innocua* (7). Identified as Listeria genus through targeted sequencing. However, *Listeria monocytogenes* does not move at 37°C and is CAMP positive. Therefore, it has been confirmed to be *Listeria monocytogenes*.

**Table 3:** The tNGS identification results of cerebrospinal.

Project	Test method	Result
Targeted gene sequencing for microbial identification	Gene sequencing method	Listeria cossartiae (NR181110.1) 99.715%

**Table 4:** The mNGS identification results of cerebrospinal fluid.

Type	Name	Sequence number	Relative abundance
G+	Listeria	36	17.29%
Composite group / Species			

Name	Sequence number	Coverage
*Listeria monocytogenes*	26	0.07%

The clear diagnosis is *Listeria monocytogenes* meningitis. Change to meropenem plus penicillin treatment, penicillin 4 million U intravenous drip, once/4 h, discontinue vancomycin and quadruple anti-tuberculosis drugs.

The gene amplification showed a single clear target band, and the sequencing results were compared by blast. The similarity with the sequences of other strains is:

1: Listeria cossartiae (NR181110.1) 99.715%.
2: *Listeria innocua* (NR116805.1) 99.644%.

Attached sequence (Figure 2C).

On the 6th day of admission, the patient’s mind improved and their mental state improved compared to before. Upon further examination of the medical history, the patient had a history of consuming sausages that had been stored in the refrigerator for a long time before the onset of the disease. We considered that this disease is related to this. At this time, the patient experienced repeated nausea and vomiting and was unable to eat. Symptomatic treatment was given to inhibit acid and protect the stomach.

On the 8th day of admission, the patient showed no further yelling or yelling. Physical examination showed resistance in the body and neck, with three transverse fingers under the chin and Kerning sign (+) and Brudzinski sign (−).

On the 18th day of admission, follow-up lumbar puncture was performed. The cerebrospinal fluid pressure is 325mmH_2_O, and the cerebrospinal fluid detection value is basically normal. At present, the patient’s condition is stable, and doctors have instructed to stop issuing major illness notices. The patient will be taken out of the ICU. However, 28th day of admission, the patient’s intracranial pressure was still very high, and pressure reduction was continued for fear of thrombosis (Figure 2A).

After treatment, the patient did not have any headaches, high fever, or restricted physical activity. The physical examination shows clear awareness, fluent language, and cooperation with the examination. The neck is soft, with Kexning sign (−) and Brudzinski sign (−). The patient stopped taking antibiotics and was discharged 52 days after recovery. After receiving full hospital treatment, the patient’s condition has improved greatly and recovering from self-care now. We compared the routine and biochemical indicators of the patient’s cerebrospinal fluid before and after treatment (Table 5). we summarized the relevant laboratory test indicators (CRP, PCT, WBC, HGB, CSF WBC, CSF Glu, CSF protein, etc.) during the patient’s hospitalization period (Figures 2D–G).

**Table 5:** The comparison of the routine and biochemical indexes of the cerebrospinal fluids before and after treatment.

Indexes	Before treatment	After 43 days of treatment
Colour	Colourless	Colourless
Transparency	Slightly turbid	Transparent
Protein	+	−
White blood cell	2.797	0.002
The absolute value of polykaryocytes (body fluids)	66	/
The absolute value of monocytes (body fluids)	34	/
Red blood cell count (body fluid)	0.000006	0.0000000
Total protein (CSF)	1.91	0.28
Glucose measurement (CSF)	0.7	2.4

## 3 Discussion and conclusion

*Listeria monocytogenes* is a bacterial infection caused by LM. In healthy individuals, infection with LM is usually asymptomatic or a mild, self-limiting disease, such as febrile gastroenteritis (8, 9). However, in older adult individuals with weakened immunity, pregnant women and newborns, or in patients with immunosuppressive therapy, *Listeria monocytogenes* can manifest as bacteremia or sepsis, central nervous system infections, etc., leading to serious or potentially fatal diseases, including sepsis or meningitis (10). It causes high mortality and high incidence rates worldwide (11).

LM meningitis is the third most common cause of community-acquired bacterial meningitis, second only to adult pneumococcal meningitis and meningococcal meningitis, but its incidence rate is relatively low, accounting for 5–10% of listeriosis cases (12). The clinical manifestations of Listeria meningitis are non-specific, mainly characterized by fever, headache, vomiting, and consciousness disorders, similar to other types of purulent meningitis. If clinical signs indicate central nervous system infection, it should not be ignored. Therefore, early diagnosis and treatment of *Listeria monocytogenes* meningitis are particularly important. According to the diagnosis and treatment guidelines, the best time to start using treatment drugs for bacterial meningitis from medical treatment is “within 1 h” (10). When the patient has meningeal irritation but fever cannot be ruled out. The patient in this case initially had neurological symptoms such as fever, headache, unclear consciousness, and vomiting; Various inflammatory indicators have increased, indicating a central nervous system infection. Cerebrospinal fluid and blood cultures have confirmed LM meningitis, and the combination of meropenem and penicillin has produced favourable clinical outcomes. Our case emphasizes the importance of identifying meningitis infection in patients with *Listeria monocytogenes* disease (13).

Early diagnosis and appropriate antibacterial treatment are the best choices for reducing mortality and disease sequelae. As is well known, intravenous injection of ampicillin or penicillin combined with intravenous injection of gentamicin is effective in treating intracranial infections in LM and is usually considered the preferred treatment. Cephalosporin drugs do not affect LM. If there are contraindications to ampicillin/penicillin, such as allergies, the use of trimethoprim/sulfamethoxazole may be considered. Adding aminoglycosides can enhance bacterial clearance, as ampicillin or penicillin alone have antibacterial effects. Cephalosporins are usually ineffective. Although vancomycin is effective against Gram-positive bacteria, its ability to penetrate eukaryotic cell membranes is limited, making it ineffective against intracellular bacteria such as Listeria. Meropenem or linezolid can also be used in patients who are allergic to ampicillin/penicillin. When clinical manifestations are difficult to distinguish from other infections, anti-infection regimens should be used to treat LM. It is recommended to use penicillin or meropenem as the initial regimen and not to use cephalosporins alone. Once confirmed as LM intracranial infection, combination therapy with penicillin or SMZ should be considered (12).

In this study, we presented a case of LM meningitis in an adult female with a clear medical history or specific risk factors (the first case discovered in our hospital in nearly 10 years). We also compiled the clinical, laboratory, and microbiological characteristics of other adults with LM meningitis (Table 6) (13–19).

**Table 6:** The characteristics of Listeria meningitis in patients aged 20–50.

Reference	Age	Gender	Clinical manifestation						CSF analysis at presentation	Duration of CSF culture or HTS and results	Antimicrobial therapy and duration	Total course of the disease (days)	Outcome
			Fever	Headache	Nausea, vomiting, diarrhea	Meningeal signs	Coma or conscious-ness disturbance	Others					
Present case	25	Female	+	+	−	+	+	Urinary incontinence	2,797/mm^3^	1 day positive	Meropenem 12 days, Penicillin 39 days	52 days	Recovered
Jamal (14)	25	Male	+	+	Vomiting +	+	−	/	933/mm^3^	2 days	Ampicillin+gentamicin9days	10 days	Recovered
Ma (15)	30	Male	+	+	Diarrhea+	+	−	Convulsions diplopia	1706/mm^3^	12 days positive	Compound sulfamethoxazole 21 days + 14 days	65 days	Recovered
Li (16)	37	Male	+	+	−	+	+	/	10,000/mm^3^	2 days positive	vancomycin 2 days, Meropenem 2 days, Rifampicin 12 days	14 days	Death
Parihar (17)	37	Male	+	+	Diarrhea +	−	−	/	847mm^3^	20 days	Amoxicillin 10 days, Gentamicin 10 days	30 days	Recovered
Giménez-Muñoz (18)	40	Male	+	+	−	−	−	/	55/mm^3^	/	Azithromycin+2 days Amoxicillin clavulanic acid 2 days, Ceftriaxone Unknown	21 days	Death
Magiar (19)	45	Male	−	+	−	−	−	diplopia	100/mm^3^	4 days positive	ampicillin 24 days vancomycin 17dys	21 days	Recovered
Cao (20)	50	Male	+	+	Vomiting +	+	+	/	835mm^3^	6 days positive	Ceftriaxone, ampicillin, Amikacin	Unknown	Satisfactory recovery

LM is the third most common pathogen detected in adult bacterial meningitis. In our review, adult LM meningitis patients (ranging from 20 to 50 years old) and susceptible groups of LM patients cases exhibited similar signs and symptoms to meningitis caused by other reasons: fever, headache, neck stiffness, altered mental state, and neurological deficits. The main transmission route of LM infection is the digestive tract. As shown in Table 6, most patients with LM infection have symptoms such as fever and headache, and some have gastrointestinal symptoms (13–19). Fever and headache are non-specific symptoms, and these patients usually do not have a clear history of food intake contamination. Some patients with LM encephalitis exhibit mild symptoms and have good treatment responses (13). However, the disease is invasive, just like the patients we mentioned earlier, with rapid progression and a record of rescue efforts. LM encephalitis develops rapidly, often accompanied by fever, cough, sputum production, difficulty breathing, and systemic symptoms. If a patient experiences delayed diagnosis, the mortality rate is extremely high, and the clinical manifestations of fever affecting the brainstem must be suspected to be Listeria infection (17). LM transverse encephalitis typically has a biphasic course with non-specific prodromal symptoms such as dizziness, headache, discomfort, and vomiting, followed by limb numbness, mild hemiplegia, decreased sensation, and changes in consciousness. In this case, no abnormalities were found in the CSF culture of the patient. Through high-throughput genome sequencing for CSF pathogen detection, the LM gene sequence was discovered, which led to a diagnosis and saved the patient’s life (19). Because LM can cross the blood–brain barrier and enter the central nervous system, infection can quickly spread to the patient’s brain and be fatal. The mortality rate of LM infection is relatively high. The initial experience of antibiotic vancomycin treatment led to patient death due to delayed treatment (15). If the use of antibiotics can be adjusted in a timely manner, there may be good results. Case reports of LM meningitis in healthy individuals with normal immune function and *Listeria monocytogenes* meningitis in adults are rare, and they may exhibit atypical influenza-like diseases or be completely asymptomatic. The patient presents with symptoms such as headache and fever, and ampicillin must be part of empirical treatment. Imaging (MRA) excluded cerebrovascular pathology and diagnosed it as the cause of infection. A large proportion of young patients with normal immune function and no triggers are diagnosed are diagnosed with *Listeria monocytogenes*, which may be secondary to food contaminated with high levels of *Listeria monocytogenes*. Therefore, it is worth noting that the absence of meningeal symptoms does not rule out Listeria meningitis.

Case reports of LM meningitis in healthy individuals with normal immune function and monocytic Listeria meningitis in adults are rare, and they may exhibit atypical flu-like diseases or may be completely asymptomatic. This case showed symptoms such as headache and fever (17). This case reminds us that clinical manifestations of fever involving the brainstem must be suspected of Listeria infection, so ampicillin must be part of empirical treatment. In addition, if patients experience symptoms such as diplopia, it should also be taken into consideration. Therefore, it is worth noting that the absence of meningeal symptoms does not rule out Listeria meningitis. In the literature, there are studies reporting hyponatremia, which is a common finding in patients diagnosed with bacterial meningitis, especially those caused by LM. In this reported case, the patient developed severe hyponatremia of 126.5 mmol/L. On the third day of admission, as the patient’s symptoms improved, the condition improved to 138 mmoL/L and only mild intravenous (iv) fluid therapy was administered. We presented this case with the aim of raising awareness of bacterial meningitis in adult normal patients who may experience atypical clinical symptoms due to LM and confirming that penicillin is a feasible treatment option for LM meningitis patients, to achieve good results. However, since this is an analysis of a case report, its reference value for treatment is somewhat limited.

In conclusion, the incidence rate of LM meningitis is extremely low, and there is no standardized treatment plan at present. Strengthening food hygiene and safety education and avoiding food infections are important measures to prevent LM infection. In clinical practice, patients with purulent meningitis, especially those with brain involvement, should be alert to possible LM infections. It is crucial to complete CSF and blood microbiology examinations as early as possible and prioritize neuroimaging evaluation. Priority should also be given to disease control, and then antibiotic regimens should be adjusted based on pathogen identification and drug sensitivity testing. It is recommended to use meropenem and ampicillin for empirical treatment. Early diagnosis and treatment can improve prognosis.

## Data Availability

The raw data supporting the conclusions of this article will be made available by the authors, without undue reservation.
